# CRISPR-MBTF: a multi-branch transformer fusion framework for CRISPR-Cas9 off-target prediction

**DOI:** 10.1093/bib/bbag216

**Published:** 2026-06-06

**Authors:** Ali Jahangiri-Sisakht, Leila Safari, Roghayyeh Alipanahi

**Affiliations:** Department of Computer Engineering, University of Zanjan, Zanjan 45371-38791, Iran; Department of Computer Engineering, University of Zanjan, Zanjan 45371-38791, Iran; Faculty of Pharmacy, Tabriz University of Medical Sciences, Golgasht Street, Tabriz, 5166/15731, Iran

**Keywords:** deep learning, CRISPR-Cas9, off-target prediction, guide RNA design, bioinformatics, computational biology

## Abstract

Clustered regularly interspaced short palindromic repeats and CRISPR-associated protein 9 (CRISPR-Cas9) is a revolutionary genome editing technology derived from a bacterial adaptive immune system that uses a single guide RNA (sgRNA) to direct the Cas9 enzyme to specific DNA sequences for precise genetic modifications. Its ease of use and efficiency has accelerated advancements in genetic research and therapeutic development. However, unintended cleavage at off-target sites remains a significant concern, limiting the safety and broader applicability of CRISPR-based editing. Accurate computational prediction of off-target locations is therefore essential to mitigate potential risks and improve experimental design. In this study, we introduce CRISPR multi-branch transformer fusion (CRISPR-MBTF), a novel deep learning-based framework employing a multi-branch Transformer architecture combined with an attention-based fusion mechanism to model the intricate biological context influencing CRISPR activity. By capturing subtle sequence patterns and contextual dependencies, our model achieves enhanced predictive performance compared to existing approaches. Additionally, interpretability analyses uncover biologically meaningful patterns and highlight influential sequence regions, offering valuable insights into the determinants of CRISPR specificity. This work presents a robust and interpretable tool to support the design of safer and more effective genome editing strategies.

## Graphical Abstract

**Graphical Abstract:**
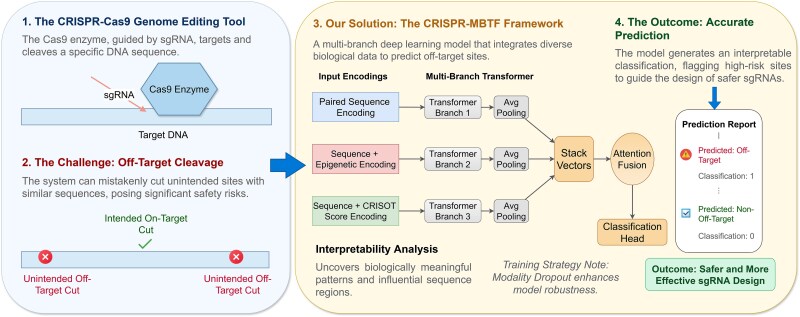

## Introduction

The clustered regularly interspaced short palindromic repeats and CRISPR-associated protein 9 (CRISPR-Cas9 system) has emerged as one of the most transformative tools in molecular biology and genetic engineering. Originally discovered as part of the adaptive immune defense in bacteria and archaea, it employs sgRNA to direct the Cas9 endonuclease to specific genomic loci, enabling precise double-stranded breaks. Owing to its simplicity, efficiency, and versatility, CRISPR-Cas9 has revolutionized therapeutic genome editing, functional genomics, and biotechnology [1–3].

Despite its precision, the CRISPR-Cas9 system can exhibit off-target activity, cleaving sequences similar but not identical to the intended target. Such unintended edits may lead to significant biological consequences, particularly in therapeutic applications where maintaining genomic integrity is critical. Predicting these off-target sites accurately remains challenging due to factors like sequence similarity, chromatin accessibility, and mismatch tolerance [4–6].

To overcome this, computational models have become increasingly vital for optimizing single guide RNA (sgRNA) design, selecting target sites, and evaluating off-target risks. Among these, deep learning (DL) has proven especially powerful, extracting complex patterns from large-scale biological datasets. Advanced architectures such as convolutional neural networks (CNNs), recurrent neural networks (RNNs), and Transformers have demonstrated strong performance in modeling the intricate, non-linear relationships between sgRNA and DNA sequences [7, 8].

Unlike traditional rule-based or feature-engineered methods, DL-based models learn directly from raw sequence data, enabling the discovery of subtle sequence motifs and contextual dependencies that influence sgRNA activity and specificity. This data-driven approach has significantly enhanced the accuracy and robustness of off-target site prediction, contributing to safer and more precise genome editing [8–11].

A critical factor in DL-based CRISPR off-target prediction is the encoding of biological sequences for neural network input. Since models operate on numerical data, raw nucleotide sequences must be transformed into representations that preserve biologically meaningful features. Traditional one-hot vector encoding techniques offer simplicity but often lack the ability to convey contextual, structural, or biochemical properties inherent to DNA sequences. In contrast, more advanced strategies aim to capture the underlying dependencies and sequence-function relationships that influence CRISPR activity. The effectiveness of a model is highly dependent on the quality of these encodings, as they directly impact the model’s ability to recognize subtle sequence variations and generalize to unseen data, both of which are essential for accurate off-target effect prediction [8, 12–14].

The challenge of off-target activity in CRISPR-Cas9 systems has driven the development of computational prediction methods, evolving from simple rule-based systems to complex multimodal DL frameworks. Early methods used rule-based mismatch scoring, such as the Massachusetts Institute of Technology (MIT) and cutting frequency determination scores, which assigned position-specific penalties to sgRNA–DNA mismatches. These scores quantified positional mismatch tolerance relative to the Protospacer Adjacent Motif (PAM), penalizing mismatches proximal to the PAM more heavily [15, 16].

While interpretable and efficient, these models had critical limitations. They could not capture non-linear, context-dependent patterns, making them ineffective in complex scenarios involving bulges, insertions, or clustered mismatches. Moreover, they were insensitive to sequence-specific features such as nucleotide identity and local GC content and generalized poorly across species or cell types because they omitted epigenetic and chromatin accessibility factors [17].

To address these limitations, classical machine-learning techniques such as support vector machines (SVMs), random forests, and gradient boosting models were introduced. These approaches, exemplified by tools such as Elevation [18], demonstrated improved predictive power by incorporating a broader set of sequence-based and thermodynamic features. However, these models still relied on engineered features and struggled to generalize to unseen sequence contexts or novel cell types.

The advent of DL enabled automated feature extraction from raw nucleotide sequences, overcoming the constraints of manual feature engineering. Early neural-network models like DeepCRISPR [19], DeepCas9 [20], and CRISPR-Net [21] employed CNNs and RNNs to process sgRNA and DNA sequences. Although they achieved superior performance, these models treated sequences as independent channels using naïve one-hot encodings, failing to explicitly model base-pair interactions.

To overcome the limitations, recent DL frameworks adopted pairwise encoding strategies that explicitly represent base-pair interactions. CRISPR-IP [14] utilized position-aware mismatch matrices that preserved spatial and mismatch-type information, enabling hybrid CNN-BiLSTM-attention architectures to extract local and global sequence features. In a different approach, CRISPR-DIPOFF [7] applied four-channel one-hot encodings independently to the sgRNA and target sequences and combined them using an element-wise logical OR operation to form a joint representation that reflects nucleotide-level complementarity. Then, this pairwise interaction encoding is processing by bidirectional RNNs to capture long-range dependencies across the aligned sequences. Additionally, CRISPR-DIPOFF incorporated integrated gradients to improve interpretability, revealing distinct functional sub-regions within the sgRNA seed: a mismatch-sensitive proximal segment (positions 16–20) and a distal segment (positions 11–15) exhibiting tolerance patterns independent of PAM proximity.

Beyond sequence similarity, biological factors also influence Cas9 cleavage efficiency. Recent studies highlight the importance of biological priors related to the cellular and molecular context [22]. One such factor is epigenetic regulation, particularly chromatin accessibility, which influences Cas9 binding and cleavage efficiency. Predictive models such as DeepSpCas9 [23] and DeepCRISPR [19] integrated epigenetic features including DNase-seq, ATAC-seq, and histone modification profiles, to enable cell-type-specific predictions. These approaches revealed that sites within closed heterochromatin regions exhibit markedly lower off-target activity, even when sequence complementarity is high, compared to sites located in open euchromatin.

Positional cleavage sensitivity along the sgRNA spacer is another crucial biological prior. The CRISOT [24] model incorporated experimentally derived mismatch penalty profiles that capture the asymmetric nature of Cas9 tolerance. These profiles revealed that mismatches at the 5′ end (positions 1–5) and the 3′ end (positions 15–20) of the spacer region substantially inhibit cleavage, while mismatches in the central region (positions 6–14) are more likely to be tolerated. Moreover, the effect of individual mismatch types, such as G-T wobble pairs, was shown to vary depending on sequence context and position.

In this work, we present a novel hybrid DL model for CRISPR-Cas9 off-target prediction that integrates three complementary encoding strategies. By combining sequence-based features, structural mismatch representations, and epigenetic context into a unified framework, our model aims to capture a more complete view of the factors influencing off-target activity. This design enables us to evaluate the individual and combined contributions of each encoding methods, ultimately enhancing predictive performance and offering deeper biological insights.

## Materials and methods

Building on recent advances, our proposed model, CRISPR-multi-branch transformer fusion (CRISPR-MBTF), introduces a multi-branch Transformer architecture designed to independently process three complementary input modalities: interaction of sgRNA and DNA sequences inspired form the encoding algorithm of DTMP-Prime [25]), epigenetic feature,s, and CRISOT-derived positional signals. Each modality is encoded through a dedicated Transformer branch, capturing distinct structural, contextual, and functional properties relevant to sgRNA–DNA interactions. These representations are subsequently integrated through a trainable attention mechanism, enabling the model to dynamically prioritize modalities on a per-instance basis. This dynamic weighting allows the model to adapt effectively to sequence-specific and context-dependent variability, leading to more robust and generalizable off-target predictions. Moreover, the attention mechanism enhances interpretability by revealing which biological signals are most influential in each prediction.

### Encoding strategies

To systematically evaluate how different input representations affect CRISPR-Cas9 off-target prediction, we developed three distinct encoding strategies for sgRNA-DNA sequence pairs. Each encoding captures different biological aspects relevant to Cas9 activity, including base-pair mismatches, bulges, and local chromatin context. We benchmarked the predictive performance of these approaches against established methods and, based on comparative insights, proposed a hybrid model that integrates the most informative features to enhance prediction accuracy.

#### Encoding A: Pairwise interaction encoding for sgRNA-DNA binding

**Figure 1:**
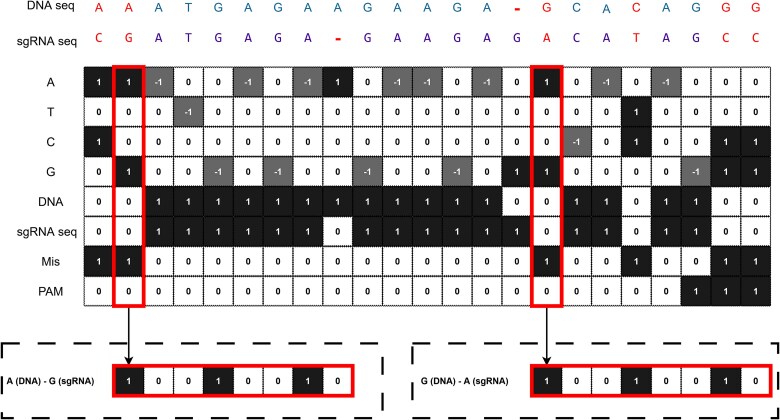
Encoding A: Pairwise interaction encoding for sgRNA-DNA binding. The 8 × 23 matrix represents sgRNA-DNA pairs, capturing nucleotide identity (rows 1–4), bulges (rows 5–6), mismatches (row 7), and PAM context (row 8). Columns corresponding to mismatches in opposite orientations are highlighted with rectangles and shown horizontally below the matrix to illustrate that the encoding does not distinguish mismatch directionality.

This strategy adapts the DTMP-Prime encoding method, originally introduced for modeling prime editing events [25], to represent sgRNA-DNA binding interactions in CRISPR-Cas9 off-target prediction. Its rule-based structure effectively captures critical features such as mismatches and bulges, which are key determinants of Cas9 targeting specificity. Unlike traditional sequence-only approaches, this encoding represents the full sgRNA-DNA interaction context, as illustrated in Fig. 1.

In our implementation, both sgRNA and DNA sequences are aligned to a fixed length of 23 nucleotides. For each pair, an 8 × 23 matrix is generated and initialized with zeros, where each column corresponds to a nucleotide position.

1. **Rows 1–4:** correspond to the four nucleotides (A, T, C, G). A value of −1 is assigned when the nucleotides at a position match, and + 1 is assigned to the respective nucleotide rows when they differ. In cases of a bulge, +1 is assigned to the row corresponding to the present nucleotide.
2. **Rows 5–6:** Row 5 denotes bulges in the sgRNA, and row 6 represents bulges in the DNA. When both nucleotides match and no gap is present, both rows take a value of 1.
3. **Row 7:** indicates a mismatch, where a value of 1 is assigned to positions with differing nucleotides.
4. **Row 8:** marks the protospacer adjacent motif (PAM) region, such as the last two or three nucleotides in SpCas9 targets (for example, NGG) [25].

Each row of the matrix encodes a biologically meaningful feature: nucleotide identity (rows 1–4), bulge presence (rows 5–6), mismatches (row 7), and PAM context (row 8). This representation enables deep learning models to capture biologically meaningful patterns, including canonical patterns and structural irregularities that influence Cas9 binding specificity and cleavage efficiency. Figure 1 illustrates an example sgRNA-DNA pair encoded using this approach.

It should be noted that the proposed encoding does not explicitly preserve mismatch directionality. Reversed mismatches (e.g., G–C versus C–G) produce identical column patterns in the encoding matrix and therefore cannot be distinguished solely from the matrix. Consequently, the representation is not lossless with respect to mismatch orientation.

#### Encoding B: Hybrid sequence-epigenetic encoding

To enhance the representation provided by Encoding A, we integrated biologically relevant epigenetic features, creating a more comprehensive representation of each sgRNA-DNA interaction site. While sequence mismatches and bulges captured by Encoding A represent structural determinants of off-target binding, chromatin state and DNA accessibility are also critical modulators of Cas9 cleavage efficiency across the genome.

We incorporated four epigenetic channels: chromatin accessibility (DNase-seq), histone modification (H3K4me3, from ChIP-seq), DNA methylation (from RRBS), and CTCF binding profiles (from ChIP-seq). This extends the original 8 × 23 Encoding A matrix into a 12 × 23 representation, with each additional row aligned nucleotide-by-nucleotide to the 23-nt target DNA sequence. All epigenetic data were obtained from the ENCODE project and processed following the schema introduced by the DeepCRISPR study [19].

Each epigenetic feature is represented as a 23-character string, where each position contains either ‘A’ (signal present, encoded as 1) or ‘N’ (signal absent, encoded as 0). For each position i:

- If both target and sgRNA share the same state (‘A’/‘A’ or ‘N’/‘N’), the position is assigned 1.
- If the states differ, the position is assigned 0.

**Figure 2:**
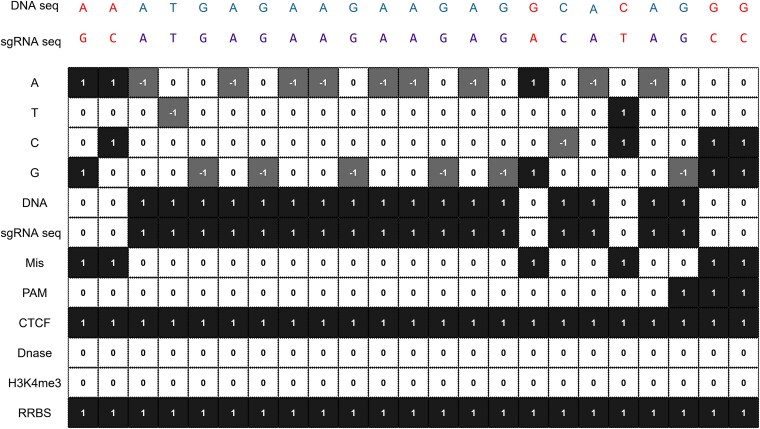
Encoding B: Hybrid sequence–epigenetic encoding integrating nucleotide features with epigenetic signals.

This encoding captures the concordance of epigenetic states at each position, allowing the model to account for the broader regulatory landscape that influences Cas9 accessibility and activity. By combining sequence features with epigenomic annotations, Encoding B offers a more comprehensive representation of each candidate site, aiming to enhance overall performance for off-target binding prediction. Figure 2 illustrates an example sgRNA-DNA pair encoded using this approach, integrating both sequence-level information and epigenetic signals.

#### Encoding C: Specificity score-enhanced encoding

**Figure 3:**
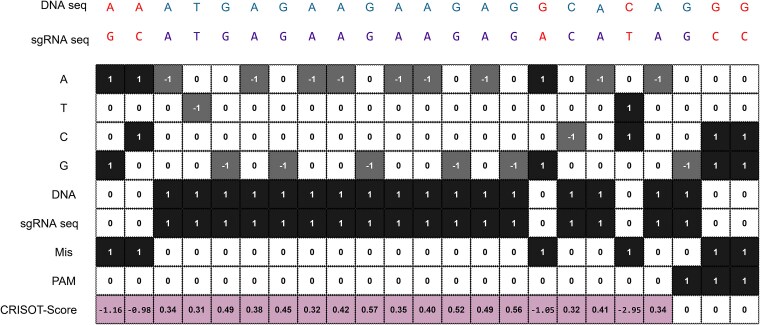
Encoding C: 9 × 23 encoding incorporating position-specific off-target likelihood scores from the CRISOT framework.

To extend the representational capacity beyond the structural determinants captured by sequence analysis and the regulatory context encoded in epigenetic features, we developed Encoding C by enriching Encoding A with position-specific off-target likelihood scores from the CRISOT framework [24] (Fig. 3). This integration enables the model to capture molecular mechanics and specificity factors that influence sgRNA-DNA binding, which may not be fully represented by sequence or chromatin features alone.

Nucleotide-level scores from the CRISOT Score module, which quantify the likelihood of off-target effects at each position within the 20-nt target site, were appended as an additional channel, transforming the 8 × 23 Encoding A matrix into a 9 × 23 representation. All CRISOT-derived scores were obtained from the publicly available CRISOT GitHub repository and used under its open-source research license. This incorporation allows the model to learn from empirically derived biophysical insights reflecting local off-target susceptibility, while remaining grounded in structural and sequence-based features.

During preliminary testing, we also evaluated the 193-dimensional sgRNA-DNA interaction fingerprints provided by CRISOT, which capture fine-grained biophysical descriptors across the 23-nt site. However, these high-dimensional vectors did not improve performance in our setting; therefore, the final Encoding C design retained only the position-specific likelihood scores, yielding a leaner and more informative representation. An example in Fig. 3 demonstrates how Encoding C integrates positional mismatch patterns with contextual sequence information.

### Model architecture

**Figure 4:**
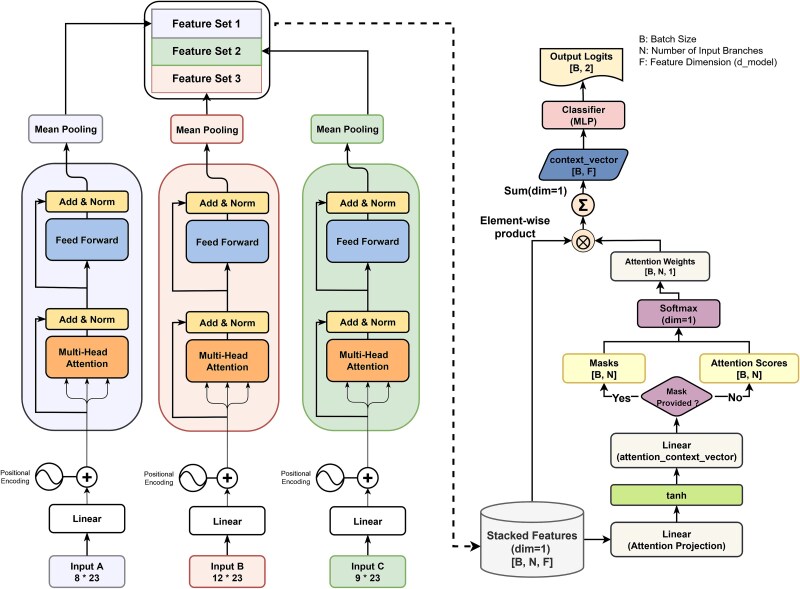
CRISPR-MBTF model architecture illustrating the integration of multiple encoding branches and attention-based feature fusion.

The proposed CRISPR-MBTF model is a multi-branch Transformer-based neural network for binary classification in CRISPR off-target prediction. A schematic overview of the architecture is shown in Fig. 4, illustrating how multiple biologically informed representations of the sgRNA-DNA pair are integrated to capture complementary aspects of sequence similarity and regulatory context.

### Per-branch feature extraction with transformer encoders

Three distinct encoding schemes are employed. Each transforms the input into a matrix reflecting different biological features such as sequence alignment, mismatch patterns, and epigenetic signals, based on domain-specific rules. These encoded representations are processed independently through dedicated Transformer encoders. Each encoder branch maps its input matrix into a high-level feature vector. This process begins with an input projection layer to match the model’s internal dimensionality ${d}_{model}$. Since standard Transformers are permutation-invariant, sinusoidal positional encodings [26] are added to provide sequence-order information:


$$ {PE}_{\left( pos,2j\right)}=\mathit{\sin}\left(\ \frac{pos}{10000^{2j/{d}_{model}}}\ \right) $$

$$ {PE}_{\left( pos,2j+1\right)}=\mathit{\cos}\left(\ \frac{pos}{10000^{2j/{d}_{model}}}\ \right) $$

The position-aware vectors are processed by self-attention and feed-forward layers. Within each branch, sequence-level information is aggregated using average pooling across the sequence dimension, yielding a fixed-length feature vector ${h}_i\in{\mathbb{R}}^{128}$ for each branch.

### Attention-based modality fusion

The outputs of the Transformer branches $\left\{{h}_1,{h}_2,{h}_3\right\}$ are stacked to form a unified multi-view representation of the input. To allow the model to dynamically weigh the importance of each branch, an attention-based fusion mechanism inspired by additive attention [27] is applied.

Each feature vector ${h}_i$ is projected into a shared attention space to compute an alignment score ${e}_i$:


$$ {e}_i={v}_a^T\ \tanh \left({W}_a\ {h}_i+{b}_a\right) $$

The learnable parameters in this formulation are:

-
${W}_a$ : a weight matrix of shape (${d}_{attn}$,${d}_{model}$) that projects the feature vector ${h}_i$(dimension ${d}_{model}=128$) into an intermediate representation of dimension ${d}_{attn}$, enabling relevance scoring in a shared space.
-
${b}_a$ : a bias vector of shape (${d}_{attn}$) that adds flexibility to the linear transformation.
-
${v}_a$ : a weight vector of shape (${d}_{attn}$), known as the ‘attention context vector’. It acts as a query to evaluate the importance of each transformed feature vector. Its transpose, ${v}_a^T$, maps the intermediate representation to a scalar alignment score ${e}_i$.

The alignment scores are normalized via the softmax function to produce attention weights that sum to one:


$$ {\alpha}_i=\frac{\mathit{\exp}\left({e}_i\right)}{\sum_{j=1}^3\mathit{\exp}\left({e}_j\right)} $$

Finally, a weighted context vector $c$ is computed to represent the overall contribution of each modality:


$$ c=\sum_{i=1}^3{\alpha}_i{h}_i $$

### Robustness via modality dropout

To improve robustness to missing data, common in biological applications, modality dropout is applied during training [28]. Random modality masking simulates scenarios where one or more branches are unavailable (e.g., missing epigenetic data). If a modality $i$ is masked, its attention score ${e}_i$ is set to a large negative value before the softmax step, forcing its attention weight ${\alpha}_i$ to zero and removing its influence on $c$. This encourages the model to perform reliably even with partial inputs.

### Classification head and training objective

The attention-weighted context vector $c$ is passed through a fully connected classification head composed of a hidden layer with nonlinear activation and dropout regularization [29], followed by an output layer producing final class predictions. The entire architecture is trained end-to-end by minimizing the cross-entropy loss:


$$ {L}_{CE}=-\sum_{k=1}^C{y}_k\log \left({p}_k\right) $$

where C is the number of classes (here, C = 2), ${y}_k$ is the ground-truth label, and ${p}_k$ is the predicted probability for class $k$. Overall, CRISPR-MBTF effectively integrates multiple feature modalities, adaptively focuses on informative signals, and remains robust in the presence of incomplete input data.

### Training the model

To train the proposed CRISPR-MBTF model, we adopted a strategy that addresses both the class imbalance inherent in CRISPR off-target datasets and the challenge of incomplete modality availability during real-world deployment. Each mini-batch was constructed to include equal numbers of positive and negative samples, preventing the model from becoming biased toward the majority class, which is a common issue in biological datasets where true off-targets are relatively rare.

The model was trained using the cross-entropy loss function and the AdamW optimizer [30] with a fixed learning rate. To mitigate overfitting and promote generalization, early stopping was applied based on the area under the precision-recall curve (PR-AUC) on the validation set. Training was terminated if no improvement in PR-AUC was observed for a defined number of epochs, and the model achieving the highest validation PR-AUC was selected as the final model.

To enhance robustness to missing data, we incorporated a modality masking strategy during training. Although all modalities were available in the training data, specific modalities were randomly masked to simulate real-world cases where certain input types (e.g., epigenetic features) may be unavailable. This approach encourages the model to learn modality-independent representations, enabling reliable performance even with partial data and making it better suited for practical CRISPR off-target prediction scenarios.

### Data collection

In this study, we trained our model using the CRISPR-Cas9 off-target dataset introduced in the DeepCRISPR study [19]. The dataset consists of sgRNA-DNA target sequence pairs labeled as 1 (off-target) or 0 (non-off-target), based on high-throughput experimental results, which serve as the ground truth for model training and evaluation.

It includes 30 sgRNAs in total, 18 from HEK293-related cell lines and 12 from K562 cells. Each sgRNA is associated to multiple potential off-target loci, creating a highly imbalanced distribution in which about 1 of every 250 loci is a true off-target [19]. This class imbalance complicates model training and requires appropriate sampling and evaluation strategies.

For the first encoding strategy, originally developed for prime editing data [25], we repurposed it for Cas9 off-target prediction. Only raw sgRNA and DNA target sequences were used, without additional features, to evaluate whether an encoding designed for another genome-editing system could generalize effectively to Cas9 applications.

In the second encoding strategy, we enriched sequence-level features with several epigenetic signals to provide biologically meaningful context. These include chromatin accessibility (DNase-seq), histone modifications (H3K4me3, ChIP-seq), DNA methylation (Reduced Representation Bisulfite Sequencing, RRBS), and CTCF binding sites (ChIP-seq). These epigenetic features were collected and integrated by the DeepCRISPR study using ENCODE data [19].

To augment representational capacity, the third encoding strategy incorporated position-specific off-target likelihood scores from the CRISOT framework [24]. These nucleotide-level values from the CRISOT-Score module quantify off-target potential along the sgRNA-DNA interface and were combined with our sequence-based encoding to form a comprehensive representation.

All CRISOT-derived scores were obtained from the publicly available CRISOT GitHub repository (https://github.com/zpliulab/CRISOT) and used under its open-source license. All encoding pipelines utilize the same dataset, ensuring fair comparisons across different feature representations in terms of their impact on model performance.

### Evaluating the model

We evaluated model performance using six standard classification metrics: Precision-Recall Area Under the Curve (PR AUC), Receiver Operating Characteristic Area Under the Curve (ROC AUC), Precision, Recall, F1 score, and Accuracy. Together, these metrics capture both ranking performance and class-wise predictive behavior, which is crucial in the highly imbalanced setting of CRISPR off-target detection [31].

Accuracy reflects overall correctness. Precision measures the proportion of predicted positives that are truly positive, helping to minimize false alarms. Recall quantifies the fraction of actual positives correctly identified, indicating the model’s sensitivity. The F1 score, the harmonic mean of precision and recall, provides a single balanced measure of both aspects.

ROC AUC assesses the model’s ability to discriminate between positive and negative classes across thresholds but can be overly optimistic in imbalanced data. In contrast, PR AUC, which plots precision against recall, offers a more informative perspective by emphasizing performance on the minority (positive) class.

Because sgRNA off-target datasets are inherently imbalanced, with far fewer true off-target sites than non-targets, PR AUC was chosen as the primary evaluation metric. This choice ensures the assessment highlights the model’s effectiveness in detecting rare positive instances, offering a stringent and biologically meaningful measure of performance.

## Results

### Experiments and results

The model’s performance was benchmarked against state-of-the-art (SOTA) methods, including CNN_Std [32], DeepCRISPR [19], AttnToMismatch [33], CnnCrispr [34], FNN_8Ch, CRISPR-IP [14], and CRISPR-DIPOFF [7]. As our work extends CRISPR-DIPOFF, we used the same dataset and baseline results to ensure consistent and fair comparison.

As shown in Table 1, the proposed multi-input Transformer achieved superior results across most evaluation metrics. It obtained the highest PR AUC, confirming its ability to distinguish true off-target sites among many negatives, which is critical for genome-editing applications. The ROC AUC also improved, indicating stronger discrimination across decision thresholds.

**Table 1:** Comparison of different models based on different performance metrics.

**Model**	**Accuracy**	**Precision**	**Recall**	**F1-Score**	**ROC AUC**	**PR AUC**
CNN_Std	0.996	0.546	0.366	0.438	0.954	0.343
DeepCRISPR	0.995	0.316	0.092	0.142	0.965	0.367
AttnToMismatch	0.987	0.166	0.512	0.251	0.965	0.309
CnnCrispr	0.996	0.500	0.748	0.599	0.987	0.678
FNN_8Ch	0.997	0.650	0.481	0.553	0.960	0.508
CRISPR-IP	0.996	**0.882**	0.115	0.203	0.987	0.610
CRISPR-DIPOFF best model	0.997	0.734	0.611	0.667	0.990	0.721
**CRISPR-MBTF(Ours)**	**0.997**	0.658	**0.779**	**0.713**	**0.997**	**0.782**

*Note*: Bold values indicate the best performance among all compared methods.

The model achieved the highest Recall and F1 score while maintaining top Accuracy. This shows better detection of true off-targets while maintaining overall performance quality. Although Precision was below the best baseline, this trade-off is acceptable since minimizing false negatives is more important than false positives in the context of biological safety.

These gains can be attributed to the model’s multi-branch Transformer architecture, which processes modality-specific inputs independently before merging them via a trainable attention-based fusion mechanism. To further improve robustness, we applied modality dropout during training, where input branches are randomly masked to simulate missing or noisy data. Ablation studies confirmed that each input contributes meaningfully and that masking enhances generalization under partial-input conditions.

**Figure 5:**
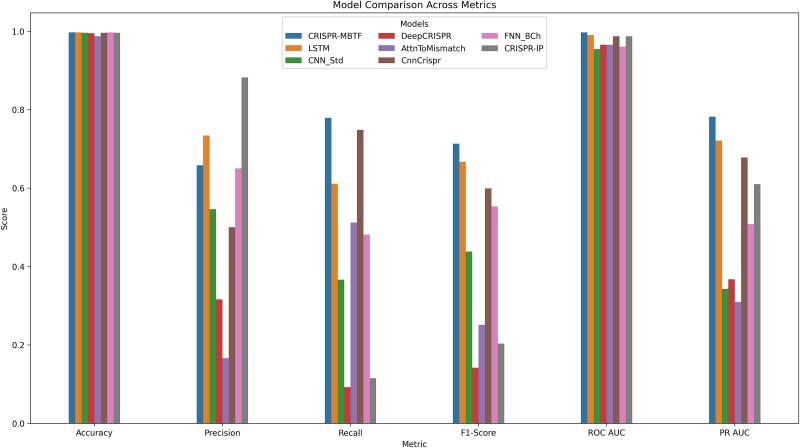
Comparison of different models based on different performance metrics. The accuracy and AUROC score are quite similar for all the models. Other metrics have varied a lot. The underlying values are reported in Table 1.

Overall, these results highlight the effectiveness of integrating biologically grounded encodings with an attention-guided fusion strategy. The model achieves SOTA PR AUC, F1 score, and related metrics while remaining resilient to incomplete data, demonstrating its utility for real-world CRISPR off-target prediction. Figure 5 illustrates these findings.

### Systematic ablation analysis

To quantify the contribution of each modality and architectural component, we conducted systematic ablation experiments by evaluating all single-branch and multi-branch combinations, as well as the effect of modality dropout. Results are summarized in Table 2.

**Table 2:** Ablation study of CRISPR-MBTF across modality configurations.

**Ablation scenario**	**Accuracy**	**Precision**	**Recall**	**F1-Score**	**ROC-AUC**	**PR-AUC**
only Encoding A	0.996	0.517	0.817	0.633	0.996	0.744
only Encoding B	0.997	0.620	0.809	0.702	0.992	0.751
only Encoding C	0.995	0.460	0.832	0.592	0.995	0.756
A + B	0.997	0.600	0.756	0.669	0.995	0.760
A + C	0.995	0.455	**0.847**	0.592	0.995	0.746
B + C	0.996	0.553	0.840	0.667	0.996	0.776
A + B + C (no modality dropout)	0.996	0.500	0.794	0.614	0.997	0.750
A + B + C (full model)	**0.997**	0.658	0.779	**0.713**	**0.997**	**0.782**
A + 193-dim CRISOT	0.997	0.618	0.718	0.664	0.993	0.736
C + 193-dim	0.997	**0.683**	0.740	0.711	0.993	0.745

*Note*: Bold values indicate the best performance among all compared methods.

Each modality independently achieved strong performance, confirming that sequence interaction patterns (A), epigenetic context (B), and position-specific CRISOT likelihood scores (C) all contain predictive signal. These results indicate that each encoding represents biologically relevant aspects of off-target prediction. However, their operating characteristics differed. The CRISOT branch exhibited the highest Recall (0.832), indicating strong sensitivity to potential off-target events, whereas the epigenetic branch achieved the highest Precision (0.62), reflecting improved discrimination in regulatory contexts.

Pairwise fusion consistently improved PR-AUC relative to single-branch models, with the B + C configuration reaching 0.776 PR-AUC, suggesting complementary contributions between regulatory and likelihood-based features. The full multi-branch model achieved the highest PR-AUC (0.782). Removing any modality resulted in a performance decline, indicating that gains are not attributable to a single dominant branch but arise from structured multimodal integration rather than feature redundancy.

We further evaluated the effect of modality dropout. Disabling random modality masking reduced PR-AUC from 0.782 to 0.75, demonstrating that modality masking improves robustness and generalization.

Finally, replacing the position-aware CRISOT branch with a 193-dimensional global CRISOT fingerprint (added as 193 new rows to Encoding A) reduced performance (PR-AUC 0.736). Combining this representation with Encoding C (C + 193) slightly decreased PR-AUC to 0.745, indicating that aggregating CRISOT information into a global vector diminishes predictive power compared to the position-aware branch. Full multi-branch fusion still achieves the best performance.

### Cross-dataset evaluation and domain shift analysis

To further assess the robustness and generalizability of CRISPR-MBTF beyond the DeepCRISPR benchmark, we conducted additional experiments using independent SITE-seq [35] and CIRCLE-seq [36] datasets. These datasets differ in experimental protocol, cell type, class imbalance ratio, and availability of epigenetic annotations.

First, we examined cross-dataset transfer under a zero-shot setting by applying the model trained on the DeepCRISPR dataset directly to external datasets without retraining. The original model was trained using all three branches (A, B, and C). However, because the external datasets do not provide epigenetic features, predictions were generated using only Branches A and C at inference time. Although modality dropout was introduced to improve robustness under partial-input conditions, the complete and systematic absence of epigenetic signals across all samples represents a distributional scenario distinct from random modality masking during training.

Under this zero-shot transfer setting, performance declined substantially compared to the model’s performance on DeepCRISPR validation data, indicating limited generalization to new datasets. This degradation likely reflects domain shift across datasets, including differences across experimental protocols, chromatin context across cell types, imbalance ratios, and epigenetic feature availability.

Second, to determine whether the observed limitation reflects architectural weaknesses or dataset-specific distribution shifts, we trained and evaluated the two-branch (A + C) CRISPR-MBTF configuration separately on each external dataset. When trained and tested on each dataset independently, the model achieved strong predictive performance on SITE-seq and CIRCLE-seq datasets (Table 3). Overall, these findings indicate that CRISPR-MBTF demonstrates strong predictive capacity on context used for training but remains challenging due to differences across experimental platforms and cell types.

**Table 3:** Performance of CRISPR-MBTF (no branch B) on SITE-seq and CIRCLE-seq datasets.

**Dataset**	**Accuracy**	**Precision**	**Recall**	**F1-Score**	**ROC AUC**	**PR AUC**
**DeepCRISPR**	0.997	0.658	0.779	0.713	0.997	0.782
SITE-seq	0.986	0.556	0.875	0.680	0.991	0.850
CIRCLE-seq	0.978	0.351	0.870	0.501	0.985	0.716

Interpretability analysis complements performance evaluation by clarifying how the model processes and prioritizes diverse input features. It bridges model performance and biological interpretability, offering insights into observed predictive patterns and guiding future improvements in model design and feature selection.

### Model interpretation via feature attribution

**Figure 6:**
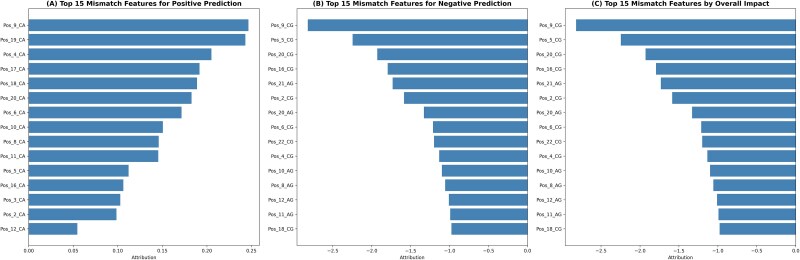
Top 15 features contributing to (A) positive predictions, (B) negative predictions, and (C) overall prediction with respect to the positive class.

To elucidate the decision-making process of our model and identify which mismatch patterns most influence off-target predictions, we applied Integrated Gradients (IG) [37] to compute feature attribution scores for all mismatch features across the 23-nt input sequence. We analyzed attributions for the positive prediction class (off-target), the negative class (non-off-target), and their overall absolute impact. Figure 6 summarizes the top 15 features contributing to (A) positive predictions, (B) negative predictions, and (C) overall prediction impact.

For the positive class, all top 15 features were CA mismatches at various positions. The most influential were CA at positions 9, 19, and 4, suggesting the model bias toward recognizing CA-type mismatches as indicative of off-target activity. Their consistent occurrence across both central and distal regions may reflect a learned pattern in which CA mismatches broadly increase the likelihood of off-target classification.

In contrast, the negative prediction class was dominated by CG and AG mismatches. The strongest negative attributions were observed for CG mismatches at positions 9, 5, and 20, followed by several AG mismatches primarily in the PAM-distal half (e.g., positions 21, 20, and 10). These negative attributions indicate that such mismatches reduce the likelihood of the model predicting a site as an off-target, potentially reflecting strong energetic destabilization or reduced mismatch tolerance in these contexts.

When evaluating overall impact (based on absolute IG magnitude), CG mismatches emerged as the most decisive contributors, regardless of whether they positively or negatively influenced predictions. Specifically, CG mismatches at positions 9, 5, and 20 had the highest attribution magnitudes, indicating the model’s confidence is highly sensitive to these sequence features.

Taken together, these attribution patterns suggest that the model has learned a biologically meaningful distinction between tolerated (e.g., CA) and non-tolerated (e.g., CG, AG) mismatches, and that positional context plays a critical role in shaping the functional impact of each mismatch type. Notably, the strong signals around positions 5–10 and 16–22 are consistent with prior studies identifying the central and PAM-distal regions as functionally important in off-target recognition [16].

### Global feature importance across modalities

**Figure 7:**
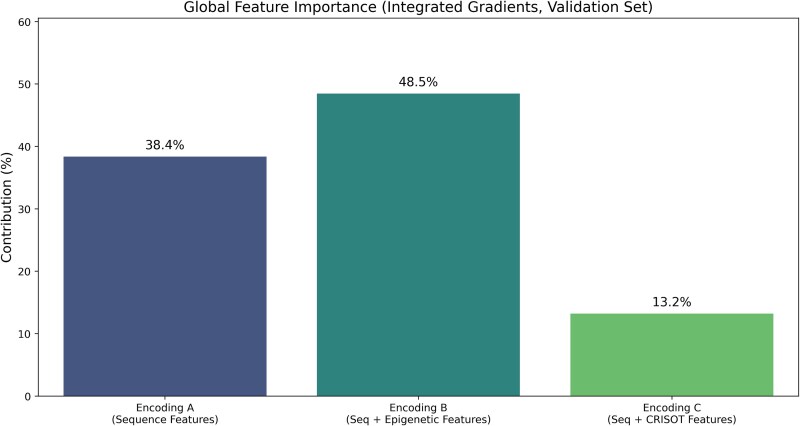
Global importance of different feature branches contributing to model predictions.

The analysis of global feature attributions reveals that epigenetic features play the most influential role in guiding the model’s predictions, surpassing both sequence-based and CRISOT-derived features. This underscores the importance of incorporating regulatory context such as chromatin accessibility and histone modifications, when evaluating off-target effects in CRISPR systems. While sequence features remain essential for capturing canonical mismatch patterns, their predictive power is notably enhanced when integrated with epigenetic information. CRISOT-derived features contribute additional complementary signals by assessing the likelihood of off-target effects, though their overall impact is comparatively smaller. Together, these findings validate the multimodal design of our architecture and emphasize the benefit of fusing diverse biological representations. These distinctions in feature importance are presented in Fig. 7.

### Model sensitivity to sequence mismatches

**Figure 8:**
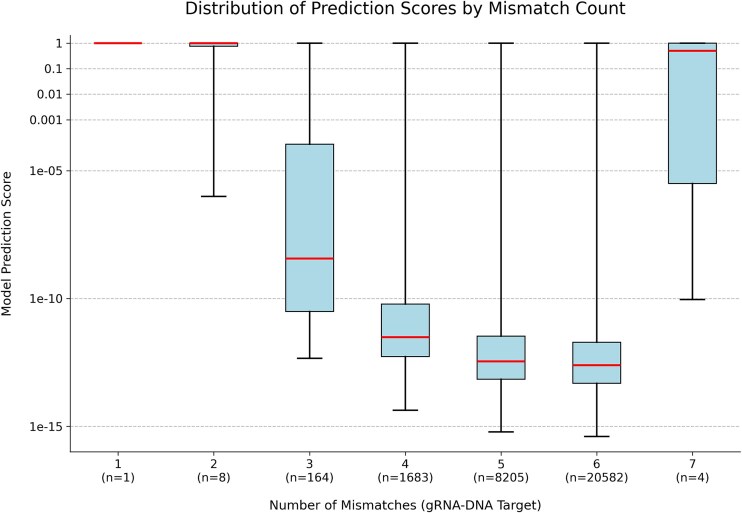
Boxplots of predicted activity scores by mismatch count (log-scaled y-axis). Scores decline sharply with increasing mismatches, though rare high-scoring outliers indicate potential potent off-targets.

To evaluate the model’s ability to discriminate based on sequence homology, we performed a mismatch sensitivity analysis. As shown in Fig. 8, there was a clear negative correlation between the number of mismatches in the DNA target and the model’s predicted off-target probability.

For targets with one mismatch (*n* = 1), the prediction score was nearly perfect (mean = 0.999985). With two mismatches (*n* = 8), the mean remained high (0.755) but exhibited greater variability (std = 0.453), indicating sensitivity not only to mismatch count but also to their positional context within the guide-target duplex. A sharp decline appeared at three mismatches (*n* = 164), where the mean dropped to 0.173. The trend continued for four, five, and six mismatches, with mean scores of 0.032, 0.006, and 0.0008, respectively.

The strong effect of multiple mismatches is further reflected in median scores, which approached zero (3.63 × 10^−9^ to 2.43 × 10^−13^) for targets with three or more mismatches, indicating that most such targets were correctly predicted as inactive. The discrepancy between mean and median values is driven by a small number of high-scoring outliers. For instance, several targets with up to six mismatches received scores >0.999 (Fig. 8). These cases likely represent potent off-targets captured by the model but overlooked by simple mismatch counting. The relatively high mean for the seven-mismatch category (0.4997) should be interpreted cautiously due to the very small sample size (*n* = 4).

Overall, this analysis confirms the model’s sensitivity to sequence mismatches and highlights its ability to detect potentially active off-targets even in the presence of substantial sequence divergence.

## Discussion

### Strengths and limitations

CRISPR-MBTF introduces key innovations that enhance CRISPR off-target prediction. Its main strength is handling heterogeneous data modalities through dedicated transformer branches, enabling the model to capture diverse aspects of guide-target interactions while remaining adaptable across datasets with different input types.

The modality-aware attention fusion allows the model to prioritize relevant branches for each prediction, improving interpretability and robustness, especially when modalities are missing or noisy. Modality dropout during training further promotes generalization, helping maintain high performance under incomplete inputs.

Quantitatively, CRISPR-MBTF outperforms CNN_Std [32], DeepCRISPR [19], CRISPR-IP, [14], and CRISPR-DIPOFF [7] across metrics such as PR AUC and Recall, reflecting strong sensitivity to true off-target sites, an essential quality in practical genome editing scenarios where false negatives can lead to severe downstream consequences.

However, the model’s multi-branch transformer design increases computational cost during training and inference, which may limit scalability for large or real-time applications. Its Precision also trails top baselines, indicating a higher false positive rate. This is a common trade-off in imbalanced classification and may require further tuning or calibration for precision-critical tasks.

Crucially, our cross-dataset evaluation highlights a limitation regarding zero-shot generalization. While CRISPR-MBTF achieves strong predictive performance when trained and tested within the same experimental platform (e.g., SITE-seq or CIRCLE-seq), its performance drops when transferring a model directly to new datasets with different experimental protocols, cell types, and missing modalities (such as absent of all epigenetic data). This indicates that domain shift remains a challenge in CRISPR off-target prediction, meaning platform-specific retraining is currently recommended for optimal real-world application.

In summary, the proposed model offers a compelling blend of performance, flexibility, and interpretability, making it a strong candidate for practical off-target risk assessment. Nonetheless, extending CRISPR-MBTF to additional cell types, species, and experimental platforms, improving computational efficiency and precision, enhancing zero-shot generalization, integrating additional genomic and epigenomic modalities, and exploring alternative encoding schemes to further optimize feature representations remain important directions for future research. Additionally, a limitation of the proposed pairwise interaction encoding is that it does not preserve mismatch directionality. Reversed mismatches (e.g., G–C versus C–G) produce identical column patterns in the encoding matrix. While this does not prevent the model from effectively predicting off-target sites, it means that the encoding is not fully lossless and could assign the same representation to biologically distinct sgRNA–DNA pairs. Finally, incorporating experimental validation will help iteratively improve predictive accuracy in practical genome editing applications.

## Conclusion

In this study, we introduced CRISPR-MBTF, a deep learning framework for CRISPR-Cas9 off-target prediction that employs a multi-branch Transformer architecture with attention mechanisms and input masking. The model integrates multiple feature types, including sequence alignment patterns, genomic context signals, and position-specific information. This design enables it to capture both the structural properties of guide-target interactions and the surrounding genomic context influencing editing outcomes.

Dedicated Transformer branches for each modality, combined with an attention-based fusion mechanism, allow the model to dynamically prioritize the most informative signals for each prediction. Robustness is enhanced through modality dropout, while feature attribution analysis provides interpretability. Our systematic ablation analysis confirms that this structured multimodal integration is essential, as performance gains arise from the complementary nature of the features rather than redundancy.

CRISPR-MBTF achieved state-of-the-art performance across multiple evaluation metrics on the DeepCRISPR benchmark, demonstrating strong ranking and classification ability on highly imbalanced datasets. Furthermore, cross-dataset evaluations on SITE-seq and CIRCLE-seq data demonstrated the model’s strong adaptability when retrained on specific platforms, though zero-shot transfer remains limited by experimental domain shift. Although its overall Precision was lower than the top baseline, it achieved the highest PR AUC, Recall, and F1 score—an acceptable trade-off in safety-critical applications where missing true off-target sites is more harmful than predicting a few false positives.

In conclusion, our results highlight the effectiveness of modality-aware Transformer architectures for CRISPR off-target prediction. The proposed framework offers a scalable and extensible foundation for future studies, including adaptation to alternative CRISPR systems and integration with experimental validation pipelines.

**Key points**

- CRISPR-MBTF: a novel multi-branch Transformer for predicting CRISPR off-target activity.
- Outperforms existing computational models for off-target site identification.
- Interpretability analysis uncovers biologically meaningful patterns of CRISPR specificity.

## Data Availability

The complete code for data processing, model training, evaluation, and interpretability analysis is publicly available on GitHub at https://github.com/JahanGit/CRISPR-MBTF. The specific version of the code used for this study (v1.0.0) and the supporting dataset are permanently archived and citable on Zenodo at https://doi.org/10.5281/zenodo.16931815 and https://doi.org/10.5281/zenodo.16919378, respectively.
